# Linking genetic and environmental factors in amphibian disease risk

**DOI:** 10.1111/eva.12264

**Published:** 2015-05-27

**Authors:** Anna E Savage, Carlos G Becker, Kelly R Zamudio

**Affiliations:** 1 Department of Ecology and Evolutionary Biology, Cornell UniversityIthaca, NY, USA; 2 Department of Biology, University of Central Florida4110 Libra Drive, Orlando, FL 32816, USA; 3 Department of Zoology, State University of Sao PauloAv. 24A No. 1515, Rio Claro, SP 13506-900, Brazil

**Keywords:** amphibian, conservation genetics, disease biology, host–parasite interactions, population genetics

## Abstract

A central question in evolutionary biology is how interactions between organisms and the environment shape genetic differentiation. The pathogen *Batrachochytrium dendrobatidis* (*Bd*) has caused variable population declines in the lowland leopard frog (*Lithobates yavapaiensis*); thus, disease has potentially shaped, or been shaped by, host genetic diversity. Environmental factors can also influence both amphibian immunity and *Bd* virulence, confounding our ability to assess the genetic effects on disease dynamics. Here, we used genetics, pathogen dynamics, and environmental data to characterize *L. yavapaiensis* populations, estimate migration, and determine relative contributions of genetic and environmental factors in predicting *Bd* dynamics. We found that the two uninfected populations belonged to a single genetic deme, whereas each infected population was genetically unique. We detected an outlier locus that deviated from neutral expectations and was significantly correlated with mortality within populations. Across populations, only environmental variables predicted infection intensity, whereas environment and genetics predicted infection prevalence, and genetic diversity alone predicted mortality. At one locality with geothermally elevated water temperatures, migration estimates revealed source–sink dynamics that have likely prevented local adaptation. We conclude that integrating genetic and environmental variation among populations provides a better understanding of *Bd* spatial epidemiology, generating more effective conservation management strategies for mitigating amphibian declines.

## Introduction

Infectious diseases are potent agents of natural selection (Darwin 1871) that impact population demography and population genetic variation, even at ecological timescales (Tishkoff and Verrelli 2003; Campbell et al. 2010). Genetic mechanisms of host resistance, host tolerance, and pathogen virulence are well known in many human (Feng et al. 2004; Barreiro and Quintana-Murci 2010) and plant (Flor 1956; Fineblum and Rausher 1995) disease systems, but far less is known about the genetic basis for evolving disease resistance in natural animal populations. Studies of wildlife populations commonly find a positive correlation between host genetic diversity and disease resistance (Meagher 1999; Pearman and Garner 2005), a pattern attributed to higher adaptive potential in genetically diverse populations or species (Frankham 2005). In contrast, other studies of host–pathogen systems detect no relationship between genetic variability of the host and prevalence of the pathogen (Ortego et al. 2007; Hawley et al. 2010). A simple relationship between host population genetics and pathogen dynamics may be unlikely, given the multitude of environmental factors that can influence disease in natural populations (Osnas and Lively 2004). Indeed, epidemiological researchers highlight the need for studies integrating genetic, spatial, and environmental processes influencing pathogen dynamics and host population genetics (Balkenhol et al. 2009; Biek and Real 2010).

Chytridiomycosis is an emerging infectious disease caused by the fungus *Batrachochytrium dendrobatidis* (*Bd*) that has caused population declines or extinction in hundreds of amphibian species worldwide (Skerratt et al. 2007; Fisher et al. 2012). Regional or population-level differences in *Bd* dynamics (Garner et al. 2006; Lips et al. 2006) are often attributed to climatic factors that affect *Bd* growth and/or persistence (Rohr et al. 2008). Although laboratory and field studies confirm *Bd* virulence is influenced by environmental variables such as latitude, elevation, precipitation, and temperature (Carey et al. 2006; Kriger et al. 2007; Brem and Lips 2008; Rohr et al. 2011), few studies have examined host genetic variability underlying chytridiomycosis susceptibility, or how the evolution of genetically resistant populations may occur across variable landscapes. To date, genetic studies of host susceptibility have focused on patterns of gene expression (Rosenblum et al. 2009, 2012; Ellison et al. 2014) and variation in innate or acquired immune genes (Woodhams et al. 2007; Savage and Zamudio 2011; Savage et al. 2014), but those data originated from laboratory experiments and did not explicitly consider population genetic composition during or after outbreaks of chytridiomycosis. To fully understand disease outcome in natural populations, we need more information about the distribution of genetic variability in natural populations, and how it relates to the evolutionary potential for disease resistance under natural environmental conditions.

The lowland leopard frog, *Lithobates yavapaiensis*, is a stream-dwelling amphibian that inhabits desert regions of southwestern North America. Historically, *L. yavapaiensis* populations expanded from northwestern Arizona into northern Mexico during the Pleistocene (Oláh-Hemmings et al. 2010). However, in recent decades, *L. yavapaiensis* has experienced population declines and range contractions (Clarkson and Rorabaugh 1989) due in part to chytridiomycosis outbreaks (Bradley et al. 2002; Savage et al. 2011). The persistence of multiple isolated populations following dramatic chytridiomycosis declines in the 1990s (Bradley et al. 2002; Schlaepfer et al. 2007; Savage et al. 2011) and *Bd* infection since at least 1974 (Hale et al. 2005) indicate that *Bd* resistance may have evolved in some populations. However, environmental variables are also important drivers of disease outcomes in this system. *Lithobates yavapaiensis* is one of several native southwestern US frog species to inhabit both geothermal and nongeothermal aquatic habitats (Bradford et al. 2004; Schlaepfer et al. 2007), and populations inhabiting ponds or streams with elevated water temperatures due to geothermal activity show significantly lower *Bd* infection prevalence than populations from nongeothermal environments (Forrest and Schlaepfer 2011; Savage et al. 2011). Thus, *L. yavapaiensis* is an ideal candidate for quantifying the evolutionary genetic consequences of an emerging infectious disease in a complex system with multiple environmental and genetic variables potentially influencing virulence.

Here, we characterize population genetic variation among *L. yavapaiensis* populations and look for associations between genetic polymorphism, environmental variation, and *Bd* disease dynamics. Chytridiomycosis varies spatially and temporally in *L. yavapaiensis* populations, causing mortality only in winter and only in some populations (Bradley et al. 2002; Savage et al. 2011). Thus, we use winter *Bd* infection and mortality estimates, multilocus host genotypes, and a suite of environmental variables to explore the relationship between host, pathogen, and environment in shaping *Bd* dynamics. We identify whether each genetic locus has a signature of neutral evolution or selection and analyze each selective category separately. Within populations, where individuals face equivalent environmental regimes, we look for disease associations between individual genetic markers and *Bd* mortality. Among populations, where individuals may face distinct environmental conditions, we consider the relative roles of host population genetics and environmental variables in predicting *Bd* dynamics. Finally, we hone in on the interplay between host, pathogen, and environment in a single locality where geothermal activity creates a disease selection gradient that drives population genetics, highlighting the fine scale of investigation that is necessary to understand the microevolutionary dynamics of host–pathogen systems. Together, our analyses provide insight into past and present interactions of host population genetics with pathogen dynamics and environmental variation in a declining amphibian species, with implications for management and conservation strategies for this and other species affected by *Bd*.

## Materials and methods

### Pathogen quantification and host genotyping in geothermal and nongeothermal environments

To quantify *Bd* dynamics, we collected epidermal swabs and observational data on *Bd*-associated morbidity and mortality from 12 *L. yavapaiensis* population localities in Arizona, USA, in January and February of 2007–2010 (winter samples from Savage et al. 2011) and January 2011 (Table S4). We pooled all samples from each locality across years and used these field demographic surveys and quantitative (q)PCR to quantify (i) *Bd* infection intensity (the number of genome equivalents, or GE) per swab (Hyatt et al. 2007), (ii) *Bd* infection prevalence (the proportion of swabbed frogs with a positive *Bd* qPCR test), and (iii) *Bd* mortality prevalence (the proportion of encountered frogs found dead or dying with visible signs of chytridiomycosis and a positive *Bd* qPCR test) (Savage et al. 2011). Most of our localities are riparian zones along tributaries of major rivers. One notable exception is the Muleshoe Ranch (MR) locality, which includes three subpopulations with variable environmental conditions: (i) The Hot Spring (MR_HS_) is a series of small interconnected pools heated by geothermal activity that are constantly at >30°C, (ii) the Secret Spring (MR_SS_) is a pond fed by another geothermal spring that is >30°C at the source but below 30°C elsewhere, and (iii) Bass Canyon (MR_BC_) is a nearby canyon stream that is not fed by a thermal spring (Forrest and Schlaepfer 2011; Savage et al. 2011). Each MR subpopulation was considered separately in all analyses because geothermal springs with elevated water temperatures can significantly alter *Bd* disease dynamics in Arizona populations of *L. yavapaiensis* (Forrest and Schlaepfer 2011).

For population genetic analyses, we collected toe tips in summer and winter of 2006–2011, preserved them in 95% ethanol, and genotyped all individuals at 14 unlinked microsatellite loci (Savage and Jaeger 2009). Many of these individuals were also swabbed for *Bd* infection metrics, presented either in this study or in Savage et al. (2011), but genetic anlyses are novel to this study. We pooled all samples from each locality across years for all subsequent analyses. We extracted genomic DNA using a 5% Chelex 100 solution (Bio-Rad Laboratories, Hercules, CA, USA) for use as templates in PCRs using previously published conditions (Savage and Jaeger 2009). Amplified products were electrophoresed on a 3730 Genetic Analyzer (Applied Biosystems, Carlsbad, CA, USA), sized using the LIZ-500 standard in the program GeneMapper v. 3.5 (Applied Biosystems), and tested for scoring errors and null alleles using MICRO-CHECKER 2.2.3 (van Oosterhout et al. 2004).

### Population genetic analyses and identification of outlier loci

We used STRUCTURE v. 2.1 (Pritchard et al. 2000a) to identify the most likely number of genetic demes (*K*) represented in our sample, allowing for admixture and assuming uncorrelated gene frequencies. We ran 20 independent runs for each value of *K*, each with 3 000 000 Markov chain Monte Carlo (MCMC) iterations after a burn-in of 1 000 000 iterations; we assessed convergence by examining summary statistics (Pritchard et al. 2000a) and used the second-order rate of change to determine the most likely value of *K* (Evanno et al. 2005).

We used GENEPOP v. 3.4 (Rousset and Raymond 1995) to calculate the observed and expected heterozygosity (*H*_O_ and *H*_E_), inbreeding (*F*_IS_), allelic richness (AR), and to test for deviations from Hardy–Weinberg equilibrium (HWE) at each locus and population locality using MCMC (1000 dememorizations, 100 batches, 1000 iterations) and Bonferroni correction for multiple tests. We estimated *D,* a measure of the fraction of allelic variation among populations (Jost 2008), for all loci and population pairs using SMOGD v. 1.2.5 (Crawford 2009) and computed pairwise *F*_ST_ values and tested for linkage disequilibrium at each locus over all populations using FSTAT 2.1 (Goudet 1995). We characterized migration among populations using BAYESASS v. 1.3 (Wilson and Rannala 2003) with a 1 000 000 burn-in, 9 000 000 iterations, and sampling every 2000 iterations. We tested for significant differences in mean population genetic parameters (*H*_O_, *F*_IS_, and AR) among populations with distinct *Bd* disease dynamics. Specifically, we compared susceptible populations (where both *Bd* infection and *Bd*-associated mortality occur), tolerant populations (where *Bd* infection occurs with no *Bd*-associated mortality), and uninfected populations (where *Bd* infection has not been detected) using Bartlett's tests, one-way anovas, and Kruskal–Wallis tests implemented in R (R Development Core Team 2008).

We used BOTTLENECK version 1.2.02 (Cornuet and Luikart 1997) to infer recent reductions or expansions of effective population size in each sampled population, excluding Upper Hassayampa (UH) due to small sample size. Specifically, we calculated for each population sample and for each microsatellite locus the distribution of heterozygosity expected from the observed number of alleles given the sample size, assuming mutation–drift equilibrium. Coalescent simulations were performed for three possible mutation models (infinite alleles model, stepwise mutation model, and the two-phase model, which allows multiple-step mutations), and resulting distributions were used to calculate average expected heterozygosity. For TPM, we set multistep mutation events to 5% and variance to 12, after Piry et al. (1999). For each mutation model, we compared observed heterozygosities and expected heterozygosities simulated 10 000 times from the allele number at equilibrium using two-tailed Wilcoxon tests, where values significantly larger than expected indicate a bottleneck, and values significantly smaller than expected indicate demographic growth. We also tested for reductions in effective population size using the *M*-ratio test implemented in M_P_Val and Critical_M (Garza and Williamson 2001). *M*, the mean ratio of the number of alleles to the range in allele size in a population sample of microsatellite loci, decreases when a population is reduced in size, and the magnitude of the decrease is positively correlated with the severity and duration of the reduction in size; thus, *M* can distinguish between populations that have been recently reduced in size and those which have been small for a long time. *M*-ratio tests require a prior value of θ (4 × effective population size × mutation rate); thus, we performed *M*-ratio tests for θ values ranging from 0.01 to 100.

Outlier loci are those that deviate from neutral evolutionary expectations and thus potentially carry a signature of natural selection. We tested for outlier loci in our sample using the Beaumont & Nichols Fdist approach (Beaumont and Balding 2004) implemented in LOSITAN (Antao et al. 2008). We simulated the neutral *F*_ST_ distribution with 100 000 iterations and a significance threshold of *P* < 0.005. We then calculated all 121 pairwise population *F*_ST_ values and compared them to population heterozygosity measures to identify *F*_ST_ outliers. Runs were performed using both the stepwise mutation model and the infinite allele model.

### Genetic and environmental disease predictors within and among populations

Within populations, we tested for associations between microsatellite genotypes and *Bd* susceptibility using STRuctured population Association Test (STRAT) (Pritchard et al. 2000b) to examine disease associations independently for each genetic deme identified in STRUCTURE (Pritchard et al. 2000a). We inferred significance of the test statistic Λ, the likelihood of association between allele frequencies and disease phenotype (1 if individual died, 0 if alive without disease signs) within demes, by comparison with 10 000 random simulations of genotype frequencies for each locus.

Across populations, we used general linear models (GLM) to test for associations of disease variables with both genetic and environmental variables. For genetic variables, we included observed heterozygosity (*H*_O_), inbreeding (*F*_IS_), and allelic richness (AR) for each population locality. For environmental variables, we extracted spatial information of nineteen bioclimatic variables of temperature and precipitation using Worldclim/Bioclim layers at 1000 m resolution (Hijmans et al. 2005; Jarvis et al. 2009) in ArcGIS 9.3.1 (ESRI 2009) for each population locality. To reduce multicolinearity issues in our GLMs (Dormann et al. 2013), we consolidated cross-correlated explanatory variables. Specifically, we consolidated 11 Worldclim/Bioclim temperature metrics (Bio1–Bio11) into two principal component (PC) axes, hereafter PC1-Temperature and PC2-Temperature. We did the same for precipitation metrics (Bio12–Bio19) and genetic (*H*_O_, *F*_IS_, AR) metrics and used the first and second PC axes from each data set (PC1-Precipitation and PC2-Precipitation and PC1-Genetics and PC2-Genetics, hereafter) in downstream model selection analyses (Figure S3). We used the scores of the first PC axis, latitude, and longitude as variables in the subsequent model selection procedures.

To test for an association between genetic and environmental factors and each disease response variable (*Bd* infection intensity, *Bd* infection prevalence, and mortality prevalence), we used a GLM model selection approach. Specifically, we included explanatory environmental and genetic PC variables and disease (*Bd* infection intensity, *Bd* infection prevalence, or mortality prevalence) as a response variable. We tested all possible models including interactions among explanatory environmental and genetic variables. Models were ranked based on Akaike information criterion (AICc), and we selected the most parsimonious model for each run.

### Measuring the potential for adaptation

To explore the dynamics of gene flow, drift, and selection among neighboring populations differing in disease epidemiology, we focused on the geothermal MR locality, where populations with different disease dynamics occur in close proximity and migration could thus potentially have large effects on the evolution of *Bd* resistance. We followed the model employed by Adkison (1995) and McCairns and Bernatchez (2008) to define the necessary demographic conditions leading to adaptive divergence among the MR populations, MR_HS_ and MR_BC_. The model is based on a numerical approximation of Slatkin's (1973) characteristic length scale of variation in gene frequency (*l*_*c*_), which defines the minimal cline distance at which populations cannot respond to environmental variation. Predictions of three alternative scenarios—genetic homogenization (H), differentiation due to random drift (R), or adaptive divergence (A)—are based on two derived variables: *β*, the ratio of migration to drift, and *k*, the ratio of the geographic scale at which selection favors a given allele (*j*) relative to *l*_*c*_ (Nagylaki and Lucier 1980). Given that MR_HS_ and MR_BC_ fall into different disease categories, we took the stringent view that these localities represent independent populations with different selection acting in each (*j *=* *1). We used a range of estimates of effective population size (*N*_*e*_) and strength of selection (*s*) to infer the conditions likely to lead to adaptive divergence (A: *β* > 1.1; *k *> 1.1), random differentiation (R: *β *< 1), or genetic homogeneity (H: *β* > 1.1; *k *<* *1) across the 95% confidence interval of estimated migration among MR_HS_ and MR_BC_.

## Results

**Figure 1:**
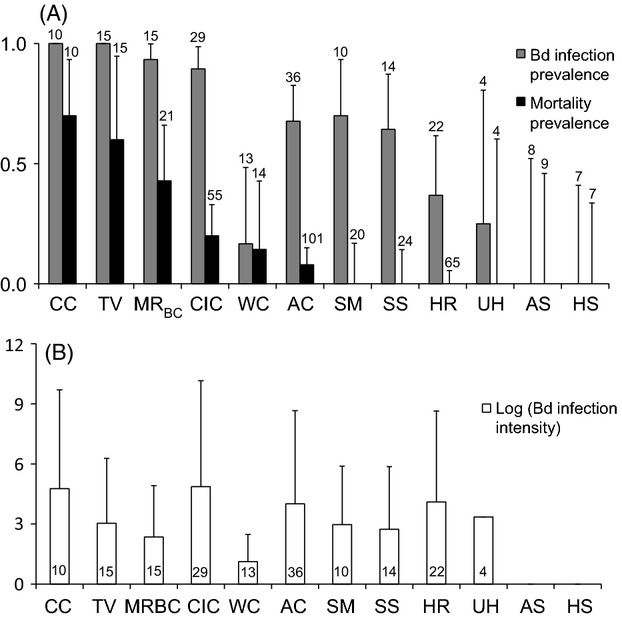
*Lithobates yavapaiensis* winter disease dynamics for 12 natural populations sampled in Arizona, USA. (A) Observed prevalence of winter *Batrachochytrium dendrobatidis* (*Bd*) infection (gray bars) and mortality (black bars) by locality, with 95% Clopper–Pearson binomial confidence intervals. Sample sizes are shown above error bars. (B) Logarithm of mean population winter *Bd* infection intensity, measured as the average number of genome equivalents recovered per animal (+SD). Sample sizes are shown inside of each bar. Locality abbreviations are as follows: AS, Aliso Spring; AC, Aravaipa Canyon; CC, Cottonwood Canyon; CIC, Cienega Creek; HS, House Spring; HR, Hassayampa River; MRBC, Muleshoe Ranch, Bass Canyon subpopulation; SM, Santa Maria River; SS, Seven Springs; TV, Tanque Verde Canyon; WC, Willow Creek; UH, Upper Hassayampa. MR Hot Springs and Secret Spring are not shown due to geothermally driven disease dynamics; see Savage et al. (2011).

We collected observational disease data and measured *Bd* infection from skin swabs for 208 *L. yavapaiensis* individuals sampled in winter (Table S4). We also genotyped 14 microsatellite markers using tissue samples from 513 *L. yavapaiensis* individuals sampled between 2006 and 2011 (mean = 46 ± 21 per site) across 12 study sites in Arizona, USA (Figs1 and 3; Table S5). Two populations were uninfected with *Bd*, six populations experienced winter chytridiomycosis mortalities, and four did not (Fig.1A). Mean *Bd* infection intensity was not significantly different between populations with and without winter mortality (Fig.1B), suggesting disease tolerance rather than resistance as a mechanism of *Bd* survival in some populations. Microsatellite markers were highly polymorphic across sampled populations (10 to 33 alleles per locus). Populations were significantly differentiated; excluding comparisons among the MR subpopulations, mean pairwise *F*_ST_ was 0.32 (range 0.17–0.60) with 90% of comparisons significant (adjusted *P *=* *0.000549) and mean *D* was 0.64 (range 0.41–0.91) with all comparisons significant (Table S1).

**Figure 2:**
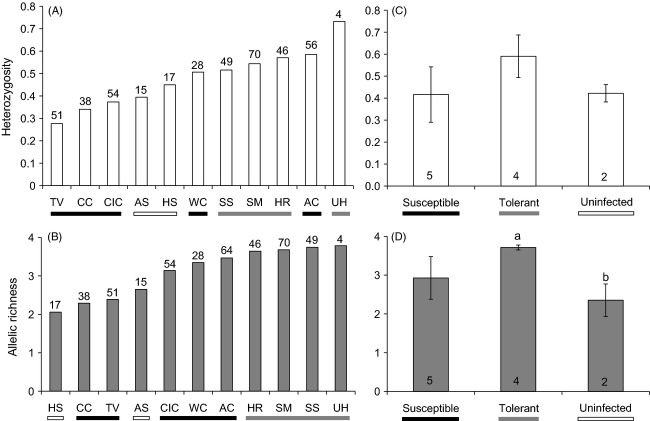
*Lithobates yavapaiensis* disease dynamics and population genetics. Mean (A) heterozygosity and (B) allelic richness for 512 individuals from 11 populations genotyped at 14 microsatellite loci are shown in ascending order with population disease status identified as susceptible (black symbols; populations with observed mortality), tolerant (gray symbols; no observed mortality), or uninfected [white symbols; *Batrachochytrium dendrobatidis* (*Bd*) not detected]. Sample sizes are shown above each bar. Mean (C) heterozygosity and (D) allelic richness across susceptible, tolerant, and uninfected population categories. Sample sizes are shown inside of each bar (*N *= number of populations). Significant differences (Student's *t*-test, *P* < 0.05) in mean values across disease categories are denoted by different lowercase letters. Population abbreviations follow Fig.1. MR is excluded due to variable environmental conditions.

*Bd*-tolerant populations showed a trend toward higher heterozygosity (*H*_O_) and allelic richness (AR; Fig.2A,B), and *Bd*-uninfected populations had the lowest measures of *H*_O_ and AR (Fig.2C,D). Mean *H*_O_ showed a nonsignificant trend toward differences between disease categories (one-way anova: *F*_2,8_ = 3.24; *P* = 0.093; Fig.2C), whereas mean allelic richness was significantly different between disease categories (one-way anova: *F*_2,8_ = 7.88; *P* = 0.013). *Post hoc* comparisons using the Tukey's HSD test indicated that mean allelic richness for *Bd*-tolerant populations was significantly different than for *Bd*-uninfected populations, but not significantly different from for *Bd*-susceptible populations (*t* = 3.75; Fig.2D). Bartlett's test did not find any evidence for significantly different variances among groups (allelic richness, χ^2^ = 2.92, *P* = 0.23; heterozygosity, χ^2^ = 0.72, *P* = 0.70); however, we also performed Kruskal–Wallis nonparametric tests to infer whether means were significantly different without assuming equal variances. Similar to anova results, mean allelic richness was significantly different between disease categories (Kruskal–Wallis test, *H* = 7.57, df = 2, *P* = 0.02), while mean heterozygosity was not (Kruskal–Wallis test, *H* = 4.32, df = 2, *P* = 0.12).

**Figure 3:**
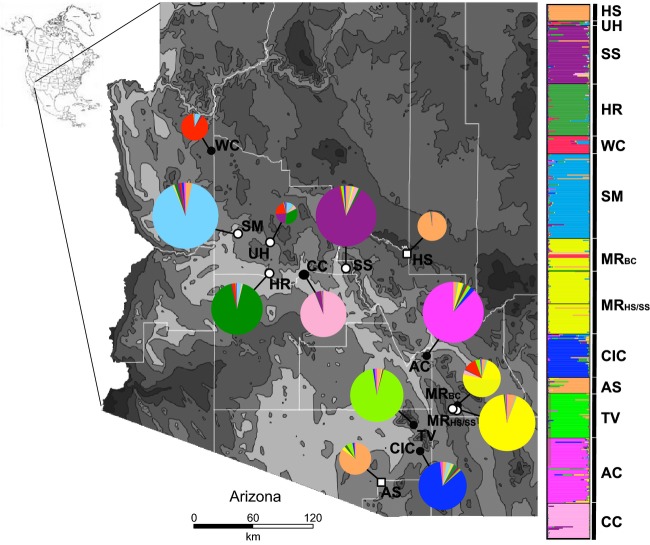
*Lithobates yavapaiensis* Structure analysis results for *K *=* *10 with individuals grouped by population locality. Left, Map of Arizona showing population localities and proportion of each population sample assigned to the ten genetic demes identified in the Structure analysis. Populations are categorized as susceptible (black circles; populations with observed mortality), tolerant (white circles; no observed mortality), or uninfected (white squares; *Batrachochytrium dendrobatidis* not detected). Circle size is proportional to the number of genotyped individuals. Right, Proportion of membership to each genetic deme for each of 512 individuals genotyped at 14 microsatellite loci. Population abbreviations follow Fig.1. The 10 genetic demes are represented as follows: AS/HS = purple; MR = light pink; SM = light blue; HR = red; SS = pink; CC = dark green; AC = light green; TV = yellow; CIC = blue; WC = tan.

Bayesian assignment revealed 10 genetic clusters that corresponded to the 12 geographic sampling localities with two exceptions: (i) Individuals from population UH showed mixed ancestry among several genetic demes, likely due to extremely small sample size (*N* = 4), and (ii) individuals from the two *Bd*-uninfected localities (HS and AS) were assigned to the same genetic deme despite the large geographic distance separating these populations (265 km) and the presence of four genetically distinct populations in the intervening region (Fig.3). Notably, these two populations were assigned to the same genetic deme for values of *K* ranging from 6 to 12, indicating a strong signal of genetic ancestry. However, both populations had among the smallest sample sizes across all populations, another potential explanation for apparent similarities. Individuals from all other geographic populations were assigned to independent genetic demes with a high average membership coefficient (*q)* of 0.89 (range 0.74–0.95; Fig.3). After identifying an outlier locus (*Ro*C110, described below), we re-ran genetic structure analyses with this locus removed due to putative non-neutral effects. Analyses excluding the outlier locus did not produce differences in deme membership or the number of inferred demes (data not shown).

We did not detect genetic signatures of population bottlenecks in any of the sampled populations using either M-ratio tests (for any θ value; data not shown) or tests of heterozygosity excess compared to simulated values under mutation–drift equilibrium (for any of the three mutation models). However, in nine of the 12 sampled populations, we detected a signature of recent demographic expansion based on a significant deficit of heterozygosity across loci under the stepwise mutation model (Table S6). Four of these populations also showed a significant pattern of demographic expansion under either the infinite alleles model (SM, a *Bd*-tolerant population, and CC, a *Bd*-susceptible population) or the two-phase model (CC, TV, and CIC, all *Bd*-susceptible populations). The three populations that did not show a pattern of demographic expansion under any model were the two thermal spring subpopulations (MR_HS_ and MR_SS_) and one of the two *Bd*-uninfected populations (HS).

Outlier locus analyses identified one of the 14 microsatellite loci as an outlier in 121 pairwise comparisons of the 11 sampled populations (Figure S1). Locus *Ro*C110 showed a pattern of directional selection with an exceptionally high *F*_ST_ value (*P *<* *0.005). All other loci fell within the expected range for neutrally evolving genetic markers. We tested the 10 identified population genetic demes for associations between microsatellite allele frequencies and two chytridiomycosis phenotypes: alive (0), for individuals observed to be alive and healthy regardless of *Bd* infection status, or dead (1), for individuals that were *Bd*-infected and found dead or dying with signs of chytridiomycosis. Across all 14 loci only *Ro*C110, the previously identified outlier locus showed a significant association between allele frequencies and *Bd* infection phenotype within genetic groups (Λ = 21.09, df = 11, *P *=* *0.009; Figure S1).

In our combined analysis of genetic variables, environmental variables, and their interactions, both genetic diversity and environmental variables were significant predictors of *Bd* infection prevalence (Table S3, Figure S2). Warmer temperatures (higher values of PC1-Temperature) were significantly associated with higher *Bd* infection prevalence (Table1), and lower population genetic variation (lower values of PC1-Genetics) was nearly significantly associated with higher *Bd* infection prevalence (*P = *0.051; Table1). In contrast, the best model explaining *Bd* infection intensity only included variation in temperature (PC1-Temperature), with higher average temperatures predicting higher *Bd* infection loads across sampled populations (*F*_1,9_ = 11.186, *r*^2^ = 0.554, *P* = 0.008). Although environmental factors were important predictors of both *Bd* infection intensity and prevalence, host genetics alone best explained mortality (*F*_1,9_ = 8.988, *r*^2^ = 0.499, *P* = 0.015). Specifically, populations with lower AR and *H*_O_ and higher *F*_IS_ (lower values of PC1-Genetics) showed significantly higher mortality rates in the field.

**Table 1:** Generalized linear model testing the effects of selected genetic and environmental factors on *Batrachochytrium dendrobatidis* (*Bd*) infection prevalence among 11 populations of *Lithobates yavapaiensis* in Arizona

Term	*β*	SE	*t* Ratio	*P*
*Bd infection prevalence*				
* Intercept*	*0.589*	*0.073*	*7.990*	*<0.001*
* *PC1-Temperature	0.108	0.039	2.710	0.026
* *PC1-Genetics	−0.136	0.059	−2.290	0.051

Whole model tests: (*F*_2,8_ = 5.173, *r*^2^ = 0.564, *P = *0.036).

**Figure 4:**
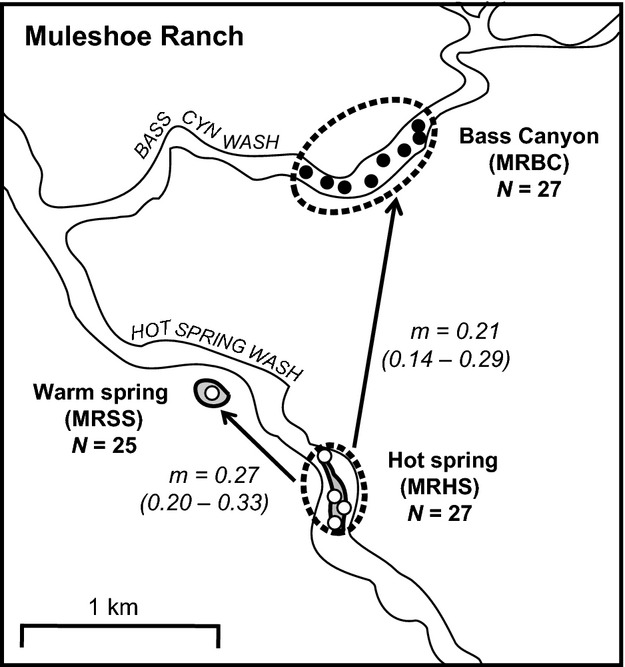
BAYESASS estimates of migration (*m*) among Muleshoe Ranch (MR) *Lithobates yavapaiensis* subpopulations (*N *= sample size). Open circles indicate sampling from localities with *Batrachochytrium dendrobatidis* (*Bd*) infection but no observed mortality. Filled circles indicate sampling from localities with *Bd* infection and observed mortality. Arrows show the presence and direction of migration. For each subpopulation, 95% confidence intervals for the proportion of individuals with immigrant ancestry are indicated in parentheses.

We detected negligible contemporary migration (*m*) among all pairs of populations (*m *=* *0–6% immigrant ancestry, mean *m *=* *0.02%, all values nonsignificant; Table S2) except among the subpopulations within the geothermal MR locality (Fig.4). Recent migration among these subpopulations was considerable, statistically significant, and unidirectional from the geothermal springs (MR_HS_) into both the pond (MR_SS_; *m *=* *27% immigrant ancestry from MR_HS_) and the canyon (MR_BC_; *m *=* *21% immigrant ancestry from MR_HS_). Migration was not detected between MR_SS_ and MR_BC_, or from either of these localities back to MR_HS_ (Fig.4). Using this information, we modeled the potential for the MR_BC_ subpopulation to evolve chytridiomycosis resistance given the high migration rate from the geothermal springs (Table2). We parameterized adaptive divergence models with a broad range of effective population sizes (*N*_e_) ranging from 10 to 10 000 individuals and included migration rates spanning the 95% confidence interval of estimated dispersal from MR_HS_ into MR_BC_ (14–29% per generation). Assuming that *Bd*-driven selection is zero at the thermal springs, the model predicts that the strength of selection (*s*; range 0–1) for chytridiomycosis resistance in the canyon must be >0.07 for the MR_BC_ frogs to adapt at the lower 95% CI of migration, independent of *N*_e_ (Table2). If migration rates are closer to the estimated mean value of 21%, *s* must be >0.11 for the canyon frogs to evolve *Bd* resistance, regardless of *N*_e_.

**Table 2:** Predicted values of effective population size (*N*_*e*_) and strength of selection (*s*) leading to adaptive divergence (A), genetic homogenization (H), or random differentiation (R) across the 95% confidence interval range of migration rates (*m*) estimated from site MRHS into site MRBC. Predictions are based on the model of Nagylaki and Lucier (1980)

*N*_*e*_	*m*	*s *=* *0.001–0.07	*s *=* *0.071–0.10	*s *=* *0.11–1
10	0.14	R	A	A
100	0.14	H	A	A
1000	0.14	H	A	A
10 000	0.14	H	A	A
100 000	0.14	H	A	A
10	0.21	R	H	A
100	0.21	H	H	A
1000	0.21	H	H	A
10 000	0.21	H	H	A
100 000	0.21	H	H	A
10	0.29	R	H	A
100	0.29	H	H	A
1000	0.29	H	H	A
10 000	0.29	H	H	A
100 000	0.29	H	H	A

## Discussion

To date, studies of *Bd* disease dynamics have identified ecological and environmental predictors of disease (Briggs et al. 2010; Becker and Zamudio 2011; Rohr et al. 2011) and have identified genetic–fitness disease associations (May et al. 2011; Savage and Zamudio 2011; Ellison et al. 2014). To our knowledge, this study is the first to combine environmental and genetic factors contributing to *Bd* disease dynamics in a single analysis, revealing that host population genetics remains a significant predictor of disease dynamics when considering the entire selective, genetic, and environmental landscape. Although environmental factors predicted the proportion and intensity of *Bd* infections in the populations, PC1-Genetics (which encapsulates 69.4% of the measured genetic variation) was the best predictor of mortality (Table S3, Figure S4). We therefore conclude that higher genetic variation measured from neutral microsatellite loci is a hallmark of lower *Bd* susceptibility in *L. yavapaiensis* and that more genetic variability within individuals and populations reduces the risk of disease susceptibility. This pattern is consistent with the ‘general effect’ hypothesis (David 1998), where heterozygosity across multiple microsatellite markers is an indirect measurement of a population's average fitness. In contrast, *Bd* infection prevalence, the most commonly measured *Bd* metric in natural populations given the considerably higher effort and cost to measure intensity and mortality, was driven by genetics, geography, and temperature across our sampled populations. We therefore caution against relying solely on infection prevalence to make inferences about the cause or effect of *Bd* in an ecosystem, as pathogen prevalence likely has numerous abiotic and biotic drivers.

Our finding that *Bd* infection intensity is determined solely by environmental factors is concordant with the high variation we observed in mean infection intensities across populations, regardless of mortality prevalence; environmental conditions dictate *Bd* growth and therefore the magnitude of infection, but some populations tolerate high infection intensities with no apparent consequences (e.g., Hassayampa River, HR) while others show mortality at fairly low infection intensities (e.g., Willow Creek, WC). The ‘10 000 Zoospore Rule’, a *Bd* infection threshold for inducing frog mortality in California's Sierra Nevada (Vredenburg et al. 2010), may therefore apply only to particular species, geographic regions, or climatic envelopes, rather than functioning as a fixed threshold for amphibian populations worldwide. Indeed, in our system, the functional consequence of infection intensity was locality-specific and could only be compared within and not among *L. yavapaiensis* populations.

Molecular adaptation to local ecological or environmental factors, including pathogens, is most commonly explored in model species with extensive genomic resources (De La Vega et al. 2002; Savolainen et al. 2013). In contrast, genetic signatures of adaptation are rarely explored in natural wildlife populations, due to the limited number of molecular markers available. Indeed, for traits under weak selection or for quantitative traits determined by multiple loci with small effects, significant patterns are unlikely to be detected from a limited set of genetic loci. However, as the strength of selection on a given trait increases, so does linkage disequilibrium (Lewontin and Kojima 1960; Schork 2002). Thus, for populations facing strong selective pressure – for example, from a disease such as chytridiomycosis that causes massive population die-offs – selection may be sufficiently strong to create genetic associations detectable by a smaller number of unlinked genomic markers. The ‘local-effects’ hypothesis, in which genotype–fitness associations result from a physical association between a neutral marker and a locus under selection (David 1998), has gained empirical support from studies showing that natural populations can show high levels of linkage disequilibrium (Yan et al. 1999) and that some loci contribute more than others to fitness associations (Hansson et al. 2004; Acevedo-Whitehouse et al. 2006). We have no direct evidence that a functional genomic region linked to outlier locus *Ro*C110 is responsible for the significant associations with mortality we detected, and further genomic work is necessary to confirm the importance of this pattern. However, for nonmodel species with large and complex genomes, inferring genetic hallmarks of disease susceptibility from a limited number of loci may be the only feasible approach and can be a useful tool for genetic management of declining populations.

The United States range of *L. yavapaiensis* has contracted precipitously in recent decades (Clarkson and Rorabaugh 1989), and *Bd*-associated mortality is a likely causative agent for these rapid declines. We thus tested for genetic signatures of population bottlenecks in each of our sampled populations. However, rather than a signal of reduced effective population size, we instead found the opposite pattern of recent population expansion in nine of 12 sampled populations (Table S6). Notably, we found the strongest support (i.e., more than one model significant) for demographic expansion in three of the five populations that are currently *Bd*-susceptible. In contrast, we found no indication of population expansion in the two *Bd*-sheltered, geothermal spring fed subpopulations (MR_HS_ and MR_SS_), or in the *Bd*-uninfected population House Spring (HS). Thus, populations that currently show *Bd*-associated declines have recently expanded, whereas populations that have not faced *Bd*-associated declines have not. Based on these patterns, we propose the following scenario: In the recent past (likely the 1970s; Hale et al. 2005), the introduction or emergence of virulent *Bd* caused a range contraction in *L. yavapaiensis* in which numerous populations were extirpated from disease. Among those populations that persisted, however, demographic expansion occurred in subsequent decades as *Bd* transitioned from epidemic to endemic (Briggs et al. 2010). This demographic expansion is more dramatic in populations that are completely *Bd*-susceptible (with environmental and/or pathogen factors enabling population persistence), compared to currently *Bd*-tolerant populations that likely held standing genetic variation for *Bd* tolerance when the pathogen first emerged and therefore did not crash as severely upon initial *Bd* outbreaks. The lack of demographic expansion in three of the four populations that have not experienced *Bd* declines (HS, MR_HS_ and MR_SS_) provides further support for this scenario. Demographic expansion was also detected in a phylogeographic analysis of *L. yavapaiensis* based on mitochondrial DNA haplotypes (Oláh-Hemmings et al. 2010), but this expansion likely took place before the last glacial maximum and thus does not pertain to *Bd*-related demographic effects. In contrast, microsatellite data provide information on more recent population demographics, and the patterns of recent expansion we recovered may reflect postdecline expansions following severe *Bd* outbreaks.

At the geothermal locality, all three subpopulations were infected with *Bd* but experienced distinct disease dynamics, most likely due to highly localized variation in water temperature (Forrest and Schlaepfer 2011). We predicted that MR_BC_ (nongeothermal) individuals were under selection for chytridiomycosis resistance, while MR_HS_ and MR_SS_ (geothermal) subpopulations were environmentally sheltered from this selective pressure. However, MR_BC_ belongs to the same genetic deme as MR_HS_ and MR_SS_ and was not significantly differentiated based on pairwise *F*_ST_ and *D* values (Table S1). This lack of differentiation exists because 14–29% of the MR_BC_ subpopulation consisted of first- or second-generation immigrants from MR_HS_ (Fig.4). Modeling the parameters of migration, drift, and selection necessary to create this scenario showed that the selection coefficient imposed by *Bd* would need to be high (*s *> 0.11) for MR_BC_ frogs to adapt given the annual influx of susceptible genotypes from MR_HS_. A selection coefficient of 0.11 corresponds to an 11% increase in fitness of individuals with the advantageous genotype when compared to individuals lacking that genotype. In genomewide studies of polymorphisms in *Drosophila*, estimated selection coefficients for single mutations range from 0.000012 to 0.02 (Jensen et al. 2008). In studies of human HIV, which faces ext reme selection pressure from drug therapies, whole-genome analyses detect selection coefficients for positively selected viral genotypes ranging from 8.0E−3 (Neher and Leitner 2010) to 0.09 (Liu et al. 2002). Thus, the threshold of 0.11 for MR_BC_ frogs is unlikely to be met, even when selection for chytridiomycosis resistance is strong. Because the evolution of host resistance is most likely a fitness trade-off (Anderson and May 1982) and MR_BC_ frogs only face punctuated selection for disease resistance in cooler months when *Bd* is most virulent (Carey et al. 2006), it is unlikely that selection will be strong enough for this population to become fixed for resistance to chytridiomycosis. Instead, a perpetual source–sink process of susceptible frogs migrating from the geothermal springs into the canyon and dying from chytridiomycosis in winter is likely to persist as long as the geothermal habitat remains. These findings underscore the critical and potentially underappreciated importance of geothermal environments for persistence of amphibians in the desert southwest since the emergence of *Bd*. In contrast, high gene flow across these environmentally distinct microhabitats prevents subpopulations from evolving disease resistance and presents a mechanism by which ecological and genetic variables interact to maintain infectious disease.

We found a single outlier locus that showed significant association to *Bd* mortality within populations, whereas only general measures of genetic diversity were significant predictors of *Bd* mortality across populations. This pattern is consistent with the lack of gene flow we detected among sampled populations; because populations are completely isolated, they evolve as independent units based upon standing measures of genetic diversity and, in the case of the outlier locus, the presence and frequency of susceptibility-associated alleles at the time of initial *Bd* emergence. Genetic similarity of the two *Bd*-uninfected populations (HS and AS) was a surprising pattern that may derive from a common genetic signature of population structure in the absence of *Bd*, although further identification and sampling of uninfected populations is necessary to draw conclusions about the precise effects of *Bd* on population genetic structure. Of note, we did not explore pathogen genetic variation in this study. Recent genetic analyses of worldwide *Bd* samples have identified genomic recombination, selection, and chromosome copy number variation (Farrer et al. 2011, 2013; Rosenblum et al. 2013) as hallmarks of virulent *Bd* strains; thus, future work incorporating fine-scale pathogen variation will likely further elucidate the epidemiology of chytridiomycosis in *L. yavapaiensis*.

Our findings demonstrate the importance of integrating genetic polymorphisms, environmental variables, and detailed measurements of disease dynamics in the field to better understand potential evolutionary responses to disease emergence in natural landscapes. Our findings may prove useful in predicting the fate of populations facing pathogen selective pressure and in designating important evolutionary lineages containing genetic variation for local adaptation to chytridiomycosis. *Bd* currently infects amphibians on every continent where it occurs (Skerratt et al. 2007), and chytridiomycosis threatens the persistence of numerous species worldwide (Fisher et al. 2012). Understanding the precise ecological and evolutionary dynamics that allow or prevent populations from persisting with *Bd* will be critical for accurate planning and implementation of species conservation efforts (Woodhams et al. 2011). For example, the hot springs locality represents a scenario where environmental sheltering allows populations to persist but also prevents adaptation to disease. Preserving geothermal habitats is therefore critical for persistence of the amphibian populations they house. Alternately, for populations with the potential to evolve *Bd* resistance, effective conservation efforts will likely entail management actions that promote genetic diversity and increase effective population sizes, as genetic diversity explained *Bd*-associated mortality in *L. yavapaiensis* but environmental factors did not (Table1). Paradoxically, the dramatic isolation of *L. yavapaiensis* populations in recent decades (Fig.3; Witte et al. 2008) has prevented ongoing gene flow from erasing local adaptation to disease, heightening the evolutionary potential for remaining populations with any *Bd-*resistant and/or *Bd-*tolerant genotypes to overcome chytridiomycosis susceptibility. Preserving and promoting genetic diversity in isolated populations is therefore likely to be the most effective management strategy to increase the long-term probability of *L. yavapaiensis* species persistence in the face of ongoing negative effects of habitat change and chytridiomycosis.

## Data archiving statement

Microsatellite data have been deposited in the Dryad repository: http://dx.doi.org/10.5061/dryad.3qk53.

## Supporting Information

**Figure S1.** *F*_ST_ versus heterozygosity for microsatellite loci and allele frequencies of the outlier locus. **Figure S2.** *Bd* disease metrics versus genetic measures for determining GLM variables. **Figure S3.** Principal Component analyses consolidating temperature [Bio1-Bio11; (A)], and precipitation [Bio12-Bio19; (B)] into two PC axes. **Figure S4.** Linear association among genetic variables [Ho, AR, FIS; (A)] and Principal Component analyses consolidating the same genetic variables into two PC axes (B). **Table S1.** Pairwise estimates of *F*_ST_ (upper) and *D* (lower) for each collection locality. All values are significant except those in italics. **Table S2.** Recent migration among populations estimated using BAYESASS. **Table S3.** Model selection for genetic and environmental factors influencing Bd infection intensity, *Bd* infection prevalence, and mortality prevalence of *L. yavapaiensis* in Arizona. **Table S4.** Sampling information for winter *Bd* measurements. **Table S5.** Sampling information for microsatellite genotyping. **Table S6.** BOTTLENECK analysis to test for significant heterozygote excess (indicating a recent population bottleneck) or deficiency (indicating demographic expansion).
