# Supplementary material for: Linking genetic and environmental factors in amphibian disease risk
Source: Evol Appl. 2015 May 27;8(6):560–72. doi: 10.1111/eva.12264 (PMC4479512; doi:10.1111/eva.12264)

**Supporting Information**

**Table S1.** Pairwise estimates of *F*_ST_ (upper) and *D* (lower) for each collection locality. All values are significant except those in italics.

|  | CC | AC | TV | AS | CIC | MR_BC_ | SM | WC | HR | SS | HS |
| --- | --- | --- | --- | --- | --- | --- | --- | --- | --- | --- | --- |
| CC | – | 0.3998 | 0.5637 | *0.565* | 0.4727 | 0.4113 | 0.377 | 0.4404 | 0.3345 | 0.2676 | 0.6044 |
| AC | 0.7131 | – | 0.3154 | 0.3184 | 0.2365 | 0.1611 | 0.2694 | 0.3005 | 0.2439 | 0.275 | 0.3281 |
| TV | 0.8252 | 0.5412 | – | *0.3934* | 0.2483 | 0.2745 | 0.4054 | 0.4793 | 0.4059 | 0.3857 | 0.4826 |
| AS | 0.8908 | 0.6740 | 0.5004 | – | *0.2752* | 0.2393 | 0.3124 | *0.3953* | *0.3359* | 0.348 | *0.4121* |
| CIC | 0.7849 | 0.5257 | 0.3373 | 0.5073 | – | 0.1781 | 0.3116 | 0.3559 | 0.3352 | 0.2986 | 0.3569 |
| MR_BC_ | 0.7636 | 0.4265 | 0.5021 | 0.5343 | 0.4465 | – | 0.2531 | 0.2561 | 0.2572 | 0.2468 | 0.3281 |
| SM | 0.7486 | 0.7345 | 0.8592 | 0.7641 | 0.7644 | 0.6991 | – | 0.2039 | 0.1585 | 0.2209 | 0.3555 |
| WC | 0.7551 | 0.7723 | 0.8849 | 0.8439 | 0.8158 | 0.6336 | 0.4916 | – | 0.18 | 0.227 | *0.4522* |
| HR | 0.6075 | 0.5974 | 0.8044 | 0.7755 | 0.8248 | 0.6836 | 0.4144 | 0.5005 | – | 0.1935 | 0.3924 |
| SS | 0.5050 | 0.7191 | 0.7840 | 0.8382 | 0.7289 | 0.6738 | 0.6099 | 0.6239 | 0.5706 | – | 0.3658 |
| HS | 0.8686 | 0.6400 | 0.5995 | 0.5555 | 0.5968 | 0.6867 | 0.7786 | 0.8403 | 0.8171 | 0.7318 | – |

**Table S2.** Recent migration among populations estimated using BAYESASS.

|  | *Migration from* | | | | | | | | | | | | |
| --- | --- | --- | --- | --- | --- | --- | --- | --- | --- | --- | --- | --- | --- |
| *Into* | CC | AC | TV | AS | CIC | MR_SS_ | MR_HS_ | MR_BC_ | SM | WC | HRP | SS | HS |
| CC | **0.990 (0.969-1)** | 0.001  (0-0.007) | 0.001  (0-0.006) | 0.001  (0-0.008) | 0.001  (0-0.005) | 0.001  (0-0.007) | 0.001  (0-0.005) | 0.001  (0-0.007) | 0.001  (0-0.006) | 0.001  (0-0.006) | 0.001  (0-0.006) | 0.001  (0-0.006) | 0.001  (0-0.007) |
| AC | 0.001  (0-0.006) | **0.982**  **(0.962-1)** | 0.004  (0-0.015) | 0.001  (0-0.006) | 0.001  (0-0.004) | 0.001  (0-0.006) | 0.004  (0-0.014) | 0.001  (0-0.005) | 0.001  (0-0.004) | 0.001  (0-0.005) | 0.001  (0-0.005) | 0.001  (0-0.006) | 0.001  (0-0.005) |
| TV | 0.001  (0-0.004) | 0.001  (0-0.004) | **0.992**  **(0.974-1)** | 0.001  (0-0.004) | 0.001  (0-0.006) | 0.001  (0-0.006) | 0.001  (0-0.005) | 0.001  (0-0.005) | 0.001  (0-0.005) | 0.001  (0-0.006) | 0.001  (0-0.004) | 0.001  (0-0.006) | 0.001  (0-0.005) |
| AS | 0.002  (0-0.015) | 0.002  (0-0.013) | 0.002  (0-0.014) | **0.977 (0.936-1)** | 0.002  (0-0.014) | 0.002  (0-0.014) | 0.002  (0-0.013) | 0.002  (0-0.015) | 0.002  (0-0.014) | 0.001  (0-0.012) | 0.002  (0-0.012) | 0.002  (0-0.011) | 0.002  (0-0.013) |
| CIC | 0.003  (0-0.013) | 0.003  (0-0.013) | 0.002  (0-0.012) | 0.052  (0-0.101) | **0.950**  **(0.931-1)** | 0.003  (0-0.014) | 0.003  (0-0.013) | 0.003  (0-0.017) | 0.003  (0-0.015) | 0.002  (0-0.013) | 0.003  (0-0.015) | 0.003  (0-0.013) | 0.004  (0-0.020) |
| MR_SS_ | 0.003  (0-0.017) | 0.003  (0-0.017) | 0.004  (0-0.017) | 0.004  (0-0.017) | 0.01  (0-0.038) | **0.679**  **(0.650-0.710)** | **0.265**  **(0.200-0.330)** | 0.004  (0-0.019) | 0.004  (0-0.017) | 0.004  (0-0.019) | 0.004  (0-0.018) | 0.004  (0-0.017) | 0.007  (0-0.030) |
| MR_HS_ | 0.001  (0-0.007) | 0.001  (0-0.006) | 0.001  (0-0.008) | 0.001  (0-0.008) | 0.001  (0-0.008) | 0.001  (0-0.007) | **0.988**  **(0.964-1)** | 0.001  (0-0.006) | 0.001  (0-0.009) | 0.001  (0-0.007) | 0.001  (0-0.008) | 0.001  (0-0.007) | 0.001  (0-0.008) |
| MR_BC_ | 0.004  (0-0.017) | 0.004  (0-0.017) | 0.022  (0-0.057) | 0.004  (0-0.006) | 0.006  (0-0.026) | 0.007  (0-0.027) | **0.213**  **(0.140-0.290)** | **0.681**  **(0.650-0.710)** | 0.004  (0-0.019) | 0.035  (0-0.077) | 0.004  (0-0.016) | 0.003  (0-0.017) | 0.008  (0-0.032) |
| SM | 0  (0-0.003) | 0.001  (0-0.004) | 0  (0-0.003) | 0.001  (0-0.004) | 0  (0-0.002) | 0  (0-0.003) | 0  (0-0.003) | 0.001  (0-0.004) | **0.994**  **(0.982-1)** | 0  (0-0.003) | 0  (0-0.003) | 0.001  (0-0.005) | 0  (0-0.003) |
| WC | 0.002  (0-0.012) | 0.002  (0-0.013) | 0.002  (0-0.012) | 0.002  (0-0.014) | 0.002  (0-0.015) | 0.002  (0-0.013) | 0.002  (0-0.011) | 0.002  (0-0.012) | 0.001  (0-0.011) | **0.980 (0.943-1)** | 0.001  (0-0.01) | 0.001  (0-0.01) | 0.002  (0-0.011) |
| HR | 0.001  (0-0.006) | 0.001  (0-0.005) | 0.001  (0-0.006) | 0.001  (0-0.005) | 0.001  (0-0.006) | 0.001  (0-0.005) | 0.001  (0-0.006) | 0.001  (0-0.006) | 0.002  (0-0.009) | 0.001  (0-0.007) | **0.987 (0.967-1)** | 0.001  (0-0.006) | 0.001  (0-0.006) |
| SS | 0.001  (0-0.005) | 0.001  (0-0.005) | 0.001  (0-0.004) | 0.001  (0-0.005) | 0.001  (0-0.004) | 0.001  (0-0.004) | 0.001  (0-0.005) | 0.001  (0-0.005) | 0.001  (0-0.006) | 0.001  (0-0.005) | 0.001  (0-0.005) | **0.991**  **(0.974-1)** | 0.001  (0-0.006) |
| HS | 0.002  (0-0.011) | 0.002  (0-0.012) | 0.002  (0-0.014) | 0.002  (0-0.011) | 0.002  (0-0.013) | 0.002  (0-0.013) | 0.002  (0-0.011) | 0.002  (0-0.013) | 0.002  (0-0.013) | 0.001  (0-0.011) | 0.001  (0-0.012) | 0.002  (0-0.014) | **0.979**  **(0.940-1)** |

**Table S3.** Model selection for genetic and environmental factors influencing Bd infection intensity, *Bd* infection prevalence, and mortality prevalence of *L. yavapaiensis* in Arizona. We compared all possible models, including one-level interactions, using Akaike Information Criterion (AICc); Five most parsimonious models are reported for each dataset.

| *Bd* infection intensity |  |  |  |
| --- | --- | --- | --- |
| **Model** | **Number** | **RSquare** | **AICc** |
| PC1-Temperature | 1 | 0.5542 | 68.9563 |
| PC1-Temperature,PC2-Genetics | 2 | 0.6033 | 72.9097 |
| PC1-Temperature,PC2-Temperature | 2 | 0.5861 | 73.3773 |
| PC1-Temperature,PC2-Precipitation | 2 | 0.5704 | 73.7853 |
| PC1-Temperature,PC1-Precipitation | 2 | 0.5637 | 73.9555 |
|  |  |  |  |
| *Bd* infection prevalence |  |  |  |
| **Model** | **Number** | **RSquare** | **AICc** |
| PC1-Temperature,PC1-Genetics | 2 | 0.564 | 40.2652 |
| PC1-Temperature | 1 | 0.2791 | 40.5585 |
| PC1-Temperature,PC2-Temperature | 2 | 0.5143 | 41.4518 |
| PC2-Temperature | 1 | 0.2001 | 41.7012 |
| PC1-Genetics | 1 | 0.1632 | 42.1983 |
|  |  |  |  |
| *Bd* mortality prevalence |  |  |  |
| **Model** | **Number** | **RSquare** | **AICc** |
| PC1-Genetics | 1 | 0.4997 | 31.1974 |
| PC1-Genetics,PC2-Genetics | 2 | 0.6217 | 33.3605 |
| PC2-Temperature | 1 | 0.3709 | 33.7166 |
| PC2-Temperature,PC1-Rainfall | 2 | 0.5927 | 34.1721 |
| PC1-Temperature,PC1-Genetics | 2 | 0.5762 | 34.6084 |

**Table S4.** Sampling information for winter *Bd* measurements. Numbers indicate the number of frogs sampled from each population across each winter (Dec – Feb). All samples were pooled across years for analyses.

| **Locality** | **Abbr.** | **UTMs**  **(Zone 12; NAD27)** | **Elevation (m)** | **2006-07** | **2007-08** | **2008-09** | **2009-10** | **2010-11** | **Total** |
| --- | --- | --- | --- | --- | --- | --- | --- | --- | --- |
| Willow Creek | WC | 276653E, 3897524N | 1590 |  | 4 | 8 |  | 1 | 13 |
| Santa Maria River | SM | 299035E, 3805275N | 970 |  | 1 | 6 | 2 | 1 | 10 |
| Hassayampa River | HR | 343507E, 3755767N | 630 | 3 | 2 | 9 | 5 | 3 | 22 |
| Seven Springs | SS | 421596E, 3758299N | 1270 |  | 1 | 6 | 6 | 1 | 14 |
| House Spring | HS | 519897E, 3756427N | 1520 |  | 7 |  |  |  | 7 |
| Aravaipa Canyon | AC | 556918E, 3637764N | 990 |  | 5 | 27 | 2 | 2 | 36 |
| Muleshoe Ranch  Hot Spring | MR_HS_ | 571674E, 3578056N | 1260 |  | 4 |  | 2 | 6 | 12 |
| Muleshoe Ranch  Secret Spring | MR_SS_ | 571192E, 3578378N | 1250 |  | 5 |  | 2 | 4 | 11 |
| Muleshoe Ranch  Bass Canyon | MR_BC_ | 570330E, 3588337N | 1270 | 1 | 4 | 1 | 5 | 4 | 15 |
| Tanque Verde Canyon | TV | 532664E, 3568974N | 1000 | 5 | 2 | 1 | 7 |  | 15 |
| Cottonwood Canyon | CC | 376736E, 3752545N | 1120 |  |  |  |  | 10 | 10 |
| Cienega Creek | CIC | 538111E, 3540254N | 1080 | 4 | 1 | 5 | 9 | 10 | 29 |
| Aliso Spring | AS | 490458E, 3494128N | 1210 |  | 3 | 2 | 3 |  | 8 |
| Upper Hassayampa | UH | 355615E, 3737783N | 680 |  | 3 | 1 |  |  | 4 |
| *Total* | *---* | *---* | *---* |  |  |  |  |  | ***208*** |

**Table S5.** Sampling information for microsatellite genotyping. Numbers indicate the number of frogs sampled from each population in each calendar year. A zero means the site was visited in that year but no frogs were sampled for genotyping, and/or no frogs were observed. Dashes means the site was not visited.

| **Locality** | **Abbr.** | **UTMs**  **(Zone 12; NAD27)** | **Elevation (m)** | **2006** | **2007** | **2008** | **2009** | **2010** | **2011** | **Total** |
| --- | --- | --- | --- | --- | --- | --- | --- | --- | --- | --- |
| Willow Creek | WC | 276653E, 3897524N | 1590 | 8 | 3 | 4 | 9 | 0 | 4 | 28 |
| Santa Maria River | SM | 299035E, 3805275N | 970 | 10 | 33 | 24 | 3 | 0 | 0 | 70 |
| Hassayampa River | HR | 343507E, 3755767N | 630 | 9 | 26 | 7 | 1 | 0 | 3 | 46 |
| Seven Springs | SS | 421596E, 3758299N | 1270 | 5 | 20 | 21 | 2 | 0 | 1 | 49 |
| House Spring | HS | 519897E, 3756427N | 1520 | -- | 12 | 1 | 4 | -- | 0 | 17 |
| Aravaipa Canyon | AC | 556918E, 3637764N | 990 | 7 | 13 | 9 | 25 | 0 | 2 | 56 |
| Muleshoe Ranch  Hot Spring | MR_HS_ | 571674E, 3578056N | 1260 | 3 | 16 | 5 | 3 | 1 | 0 | 28 |
| Muleshoe Ranch  Secret Spring | MR_SS_ | 571192E, 3578378N | 1250 | 12 | 13 | 0 | 0 | 0 | 0 | 25 |
| Muleshoe Ranch  Bass Canyon | MR_BC_ | 570330E, 3588337N | 1270 | 0 | 11 | 7 | 6 | 3 | 5 | 32 |
| Tanque Verde Canyon | TV | 532664E, 3568974N | 1000 | 11 | 17 | 8 | 15 | 0 | 0 | 51 |
| Cottonwood Canyon | CC | 376736E, 3752545N | 1120 | 10 | 19 | 0 | 0 | 0 | 9 | 38 |
| Cienega Creek | CIC | 538111E, 3540254N | 1080 | 9 | 15 | 12 | 6 | 0 | 12 | 54 |
| Aliso Spring | AS | 490458E, 3494128N | 1210 | 5 | 6 | 2 | 0 | 2 | -- | 15 |
| Upper Hassayampa | UH | 355615E, 3737783N | 680 | -- | 3 | 1 | 0 | -- | 0 | 4 |
| *Total* | *---* | *---* | *---* |  |  |  |  |  |  | ***513*** |

**Table S6.** BOTTLENECK analysis to test for significant heterozygote excess (indicating a recent population bottleneck) or deficiency (indicating demographic expansion). Results for both the Infinite Alleles Model and the Stepwise Mutation Model are shown. Significant values are in bold italics. Population UH is excluded due to small sample size.

| **Population** | **Mean Heterozygosity** | **Infinite Alleles Model 2-tailed *P*-value** | **Stepwise Mutation Model 2-tailed *P*-value** | **Two-Phase Model**  **2-tailed *P*-value** | **Heterozygote deficiency (D) or excess (E)** |
| --- | --- | --- | --- | --- | --- |
| CC | 0.5114 | ***0.029541*** | ***0.000061*** | ***0.000183*** | D |
| AC | 0.6381 | 0.903198 | ***0.000122*** | 0.172607 | D |
| TV | 0.4289 | 0.216553 | ***0.000061*** | ***0.029541*** | D |
| AS | 0.4870 | 0.855225 | ***0.016602*** | 0.42627 | D |
| CIC | 0.5980 | 0.078491 | ***0.000305*** | ***0.005371*** | D |
| MR_SS_ | 0.6447 | 0.078491 | 0.357544 | 0.267578 | n.s. |
| MR_HS_ | 0.5920 | 0.080322 | 0.127197 | 0.414307 | n.s. |
| MR_BC_ | 0.6716 | 0.951538 | ***0.000854*** | 0.541626 | D |
| SM | 0.6796 | ***0.035278*** | ***0.000427*** | 0.463135 | D |
| WC | 0.6106 | 0.057983 | ***0.016602*** | 0.390991 | D |
| HR | 0.6738 | 0.42627 | ***0.00061*** | 0.714844 | D |
| SS | 0.6835 | 0.625732 | ***0.001526*** | 0.390991 | D |
| HS | 0.3822 | 0.30127 | 0.850098 | 0.469727 | n.s. |

**Figure S1**. (a) *F*_ST_ versus heterozygosity for fourteen microsatellite loci. *F*_ST_ was measured for all 121 pairwise population comparisons and mean *F*_ST_ between each locus and all other loci are shown. The dark gray area shows the upper 99.5% confidence interval and the white area shows the lower 99.5% confidence interval with 10,000 simulations from the stepwise mutation model. One locus, *Ro*C110, fell outside of the 99.5% confidence interval and was consider an outlier, while a second locus was slightly below the 99.5% cut-off and was not included in subsequent outlier analysis (b) Allele frequencies of the outlier locus *Ro*C110 that showed significant associations with Bd mortality within genetic demes. Numbers of individuals and allele frequencies among alive and dead *L. yavapaiensis* individuals are shown for the five genetic demes that experience *Bd*-associated mortality. Each shade of grey represents a different allele, with the same color in different populations representing the same allele.


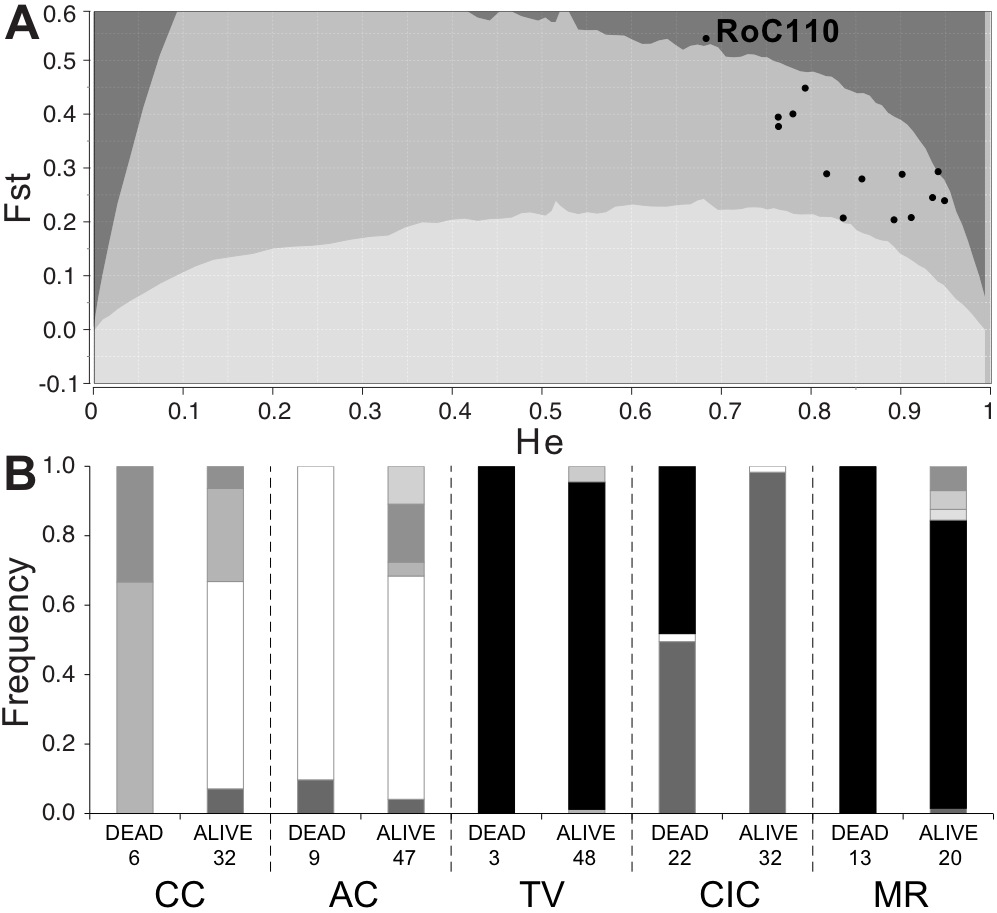


**Figure S2**. *Bd* disease metrics versus genetic measures for determining GLM variables.

**Figure S3.** Principal Component analyses consolidating temperature [Bio1-Bio11; (A)], and precipitation [Bio12-Bio19; (B)] into two PC axes.


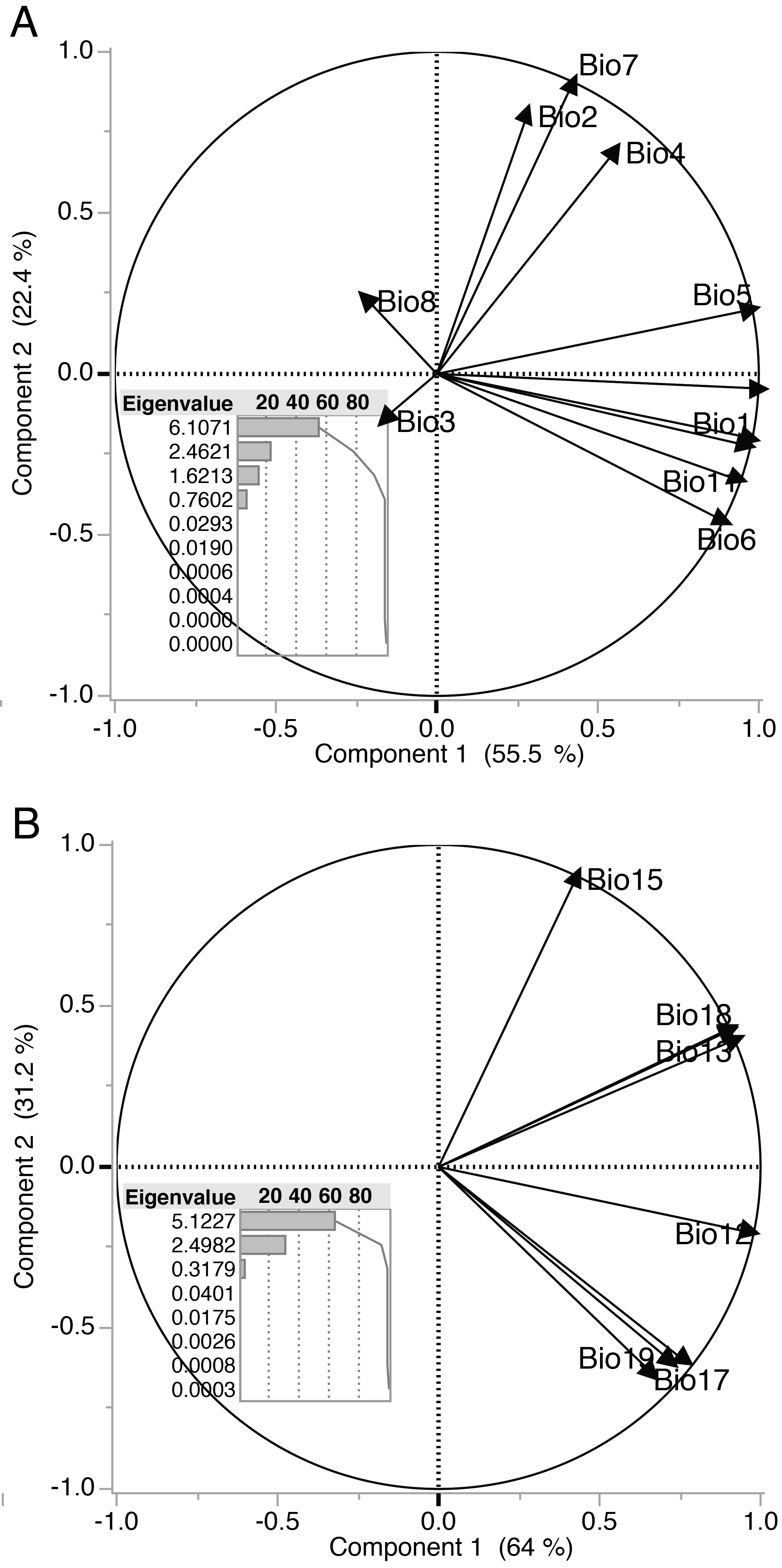


**Figure S4.** Linear association among genetic variables [Ho, AR, FIS; (A)] and Principal Component analyses consolidating the same genetic variables into two PC axes (B).


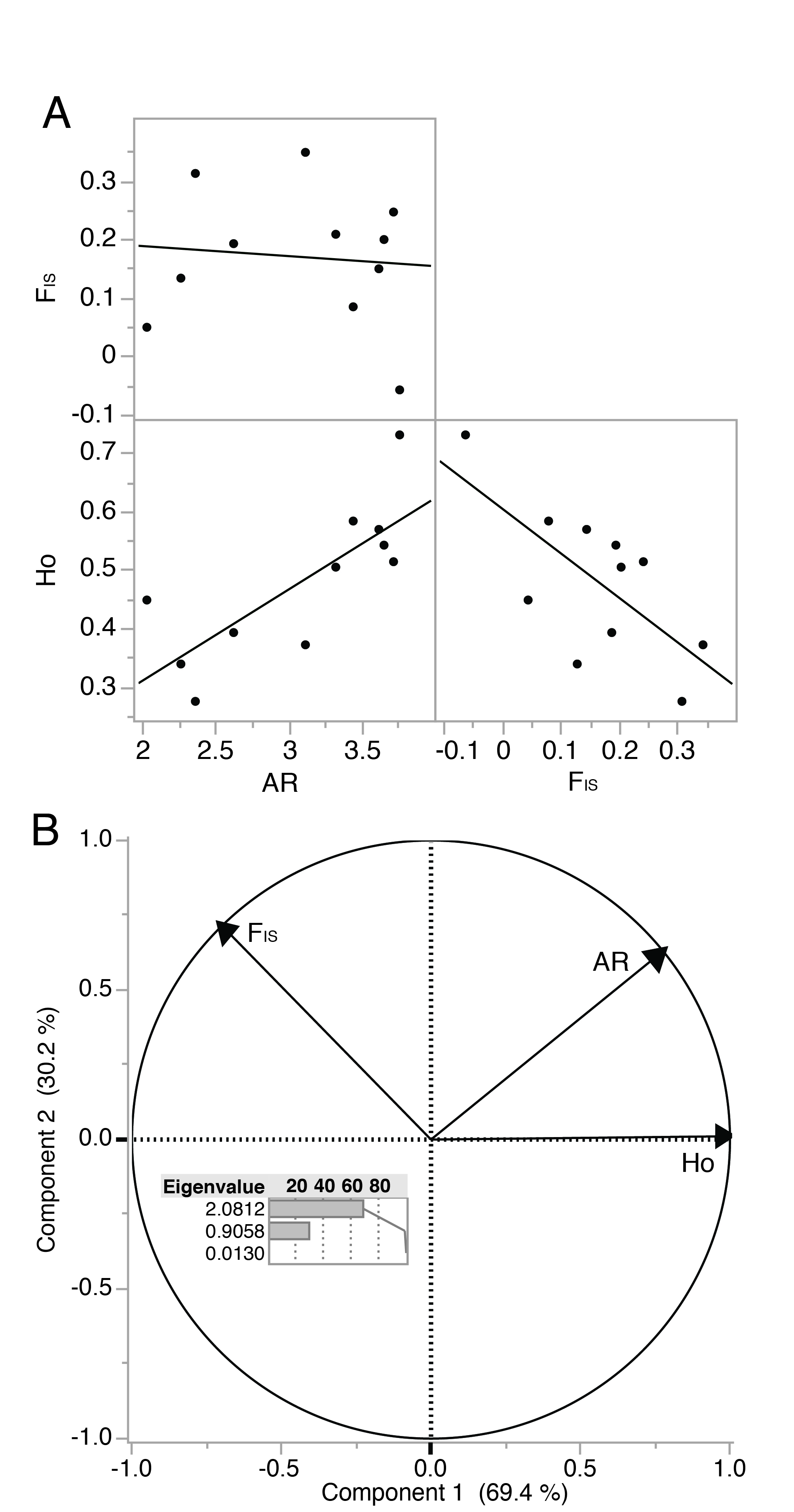

Supplement: Supplementary file 1 [file eva0008-0560-sd1.docx]
